# Association of Cardiorespiratory Fitness and Overweight with Risk of Type 2 Diabetes in Japanese Men

**DOI:** 10.1371/journal.pone.0098508

**Published:** 2014-06-04

**Authors:** Keisuke Kuwahara, Akihiko Uehara, Kayo Kurotani, Ngoc Minh Pham, Akiko Nanri, Makoto Yamamoto, Tetsuya Mizoue

**Affiliations:** 1 Department of Epidemiology and Prevention, Center for Clinical Sciences, National Center for Global Health and Medicine, Tokyo, Japan; 2 Yamaha Corporation, Shizuoka, Japan; 3 Department of Epidemiology, Faculty of Public Health, Thai Nguyen University of Medicine and Pharmacy, Thai Nguyen Province, Vietnam; College of Pharmacy, University of Florida, United States of America

## Abstract

**Objective:**

Existing evidence is limited on what extent fitness can counterbalance type 2 diabetes mellitus (T2DM) risk associated with obesity. We investigated the joint association of weight status and estimated VO_2max_, a marker of fitness, with the risk of developing T2DM among Japanese men using haemoglobin A1c and fasting glucose criterion.

**Methods:**

The present study included 3,523 male employees aged 18–61 years without diabetes who provided health check-up and fitness data in Japan in 2003–2005. We calculated hazard ratios and 95% confidence intervals for incident diabetes using the Cox regression model.

**Results:**

During a mean follow-up of 6.0 years, 199 men developed diabetes. Multivariable-adjusted hazard ratios (95% confidence interval) of diabetes were 1.00 (reference), 1.44 (1.01–2.07), and 1.48 (1.03–2.13) for the highest through the lowest tertile of fitness (P for trend  = 0.04). Additional adjustment for body mass index largely attenuated the association of fitness with diabetes. Joint analysis showed that adjusted hazard ratios of diabetes were 1.00, 1.32, 2.94, and 1.83 in normal weight high-fit men, normal weight low-fit men, overweight high-fit men, and overweight low-fit men, respectively.

**Conclusion:**

The results suggest that weight control is more important than fitness in prevention of type 2 diabetes in Japanese men.

## Introduction

The rapid increase in the prevalence of diabetes is a global public health concern [1]. Among modifiable factors, regular physical activity has been considered a cornerstone in the prevention and management of type 2 diabetes mellitus (T2DM) [2]. Accumulating evidence suggests that moderate to vigorous physical activity [3], [4], [5] can decrease the risk of T2DM. Nonetheless, since studies based on self-report of physical activity are vulnerable to reporting bias [6], studies that objectively measure physical activity would provide valuable data for the understanding of the role of physical activity in the prevention of T2DM [7].

Cardiorespiratory fitness is a health-related component of physical fitness and is a physiologic marker and objective measurement of habitual physical activity [8]. Cardiorespiratory fitness is defined as the ability of the circulatory and respiratory systems to supply fuel during sustained physical activity [9]. Estimated VO_2max_ (maximal oxygen uptake) is a measure of this type of fitness and can be easily and safely obtained from heart rates monitored under a submaximal exercise test [10]. Previous cohort studies in the US [11], [12], [13], Canada [14], and Japan [15] have shown that the cardiorespiratory fitness was inversely associated with the risk of type 2 diabetes.

As with physical activity, weight control has been a major component of type 2 diabetes prevention [16]. A meta-analysis of cohort studies [17] showed that overweight and obesity was associated with an increased risk of T2DM. It is unclear, however, whether physical fitness can counterbalance the risk of T2DM associated with obesity [18]. To date, two US reports [11], [12] from the Aerobics Center Longitudinal Study (ACLS), in which T2DM incidence was defined using fasting glucose (and self-report [11]), suggested that fitness did not eliminate the risk of type 2 diabetes associated with overweight/obesity. Evidence on this issue, however, is lacking in Asians, who may have different pathogenesis of T2DM from Caucasians [19]. Here, we examined joint associations of estimated VO_2max_ and obesity with the risk of T2DM in Japanese men using both haemoglobin A1c (HbA1c), which has been recently adopted as a diagnostic test for diabetes by the International Expert Committee [20], and fasting glucose for the identification of new type 2 diabetes cases.

## Methods

### Study design

This study is a part of the Japan Epidemiology Collaboration on Occupational Health (J-ECOH) Study, a multi-center epidemiologic study among workers from several companies in Japan. In one of the participating companies, a fitness test including measures of health-related and skill-related fitness has been performed among all eligible employees at the time of annual health check-up since 2003. Data from these fitness tests, as well as data from general health examinations between 2003 and 2011 were combined to investigate the association of physical fitness and physical activity with diabetes and other diseases. The study protocol was approved by the Ethics Committee of the National Center for Global Health and Medicine, Japan.

### Ethics statement

Prior to the collection of data, the conduct of the J-ECOH Study was announced in each company by using posters that explained the purpose and procedure of the study. Participants did not provide their verbal or written informed consent to join the study but were allowed to refuse their participation. This procedure conforms to the Japanese Ethical Guidelines for Epidemiological Research [21]; informed consent is not necessarily required for observational studies using existing data, but researchers should disclose information on the objective and the conduct of the study, and shall provide prospective subjects an opportunity to refuse inclusion of their data in the research [21]. The study protocol was approved by the Ethics Committee of the National Center for Global Health and Medicine, Japan. Most participating companies provided data in either anonymized or de-identified form, but a few other companies provided data including identifiable information, which was removed from analytic database. The data are hosted in the National Center for Global Health and Medicine. Currently, the data cannot be widely shared because the research group has not obtained permission from participating companies to provide the data on request. However, the data can be requested by other researchers for the purpose of academic, non-commercial research; inquiries and applications can be made to Department of Epidemiology and Prevention, Center for Clinical Sciences, National Center for Global Health and Medicine, Tokyo, Japan.

### Subjects

A total of 4,700 subjects (3,949 men and 751 women) aged 18 to 61 years had data on VO_2max_ between 2003 and 2005. If a subject underwent two or more tests for cardiorespiratory fitness between 2003 and 2005, the first data were considered as baseline data. Female employees were excluded from analysis because of a large gender difference in cardiorespiratory fitness level [22] and few cases of incident T2DM among women (n = 22). Of men with estimated VO_2max_ data, we excluded 290 subjects who reported a history of cancer (n = 24), cardiovascular disease (n = 63), stroke (n = 25), or diabetes or those who had fasting glucose ≥126 mg/dl (7.0 mmol/l) or HbA1c ≥6.5% (48 mmol/mol) at baseline (n = 187), or who had missing information on covariates (n = 3). Some of the excluded subjects met two or more exclusion criteria. We additionally excluded 136 subjects who did not attend any subsequent health check-ups, resulting in a total of 3,523 men aged 18 to 61 years for the present analytic cohort.

### Estimated VO_2max_ measurement

To obtain estimated VO_2max_, study participants performed an incremental endurance exercise test using a bicycle ergometer (Aerobike 900U, Combi, Tokyo, Japan) with three phases of 3 minutes duration each (a total of 9 minutes). During the first phase, the participant exercised at a workload of 30 W, and the workload was increased up to 70% of age- and sex-specific estimated maximum heart rate in the second and third phases. Subjects were asked to pedal constantly at 50 rpm. VO_2max_ was calculated from the estimated physical work capacity performed at 75% of maximal heart rate (PWC_75%HRmax_), which was obtained by creating a regression equation from heart rate monitored by an ear lobe. A validation study for a former type of the ergometer (Aerobike 500, Combi, Tokyo, Japan) showed that the PWC_75%HRmax_ is highly correlated with VO_2max_ directly measured by the Douglas bag method using an expired gas analyser ([23]; r = 0.942, P<0.001).

### General health examination

All of the subjects underwent their health examinations following an overnight fast of at least 10 hours. Body height was measured to the nearest 0.1 cm, and body weight was measured to the nearest 0.1 kg. Body mass index (BMI) was calculated as weight in kilograms divided by squared height in meters. Blood pressure was measured in a sitting position using a mercury manometer (Kenzmedico co. Ltd., Saitama, Japan) after several minutes of rest. Personal and family history of disease and health-related lifestyle (smoking, alcohol, physical activity, and sleep) were ascertained using a standard questionnaire. Biochemical measurements included plasma glucose and HbA1c levels. Blood glucose concentration was assayed by the hexokinase-UV method using Quick-auto II GLU-HK (Shino-Test Corp., Tokyo, Japan), and HbA1c was measured with a latex agglutination immunoassay using the Determiner HbA1c kit (Kyowa Medex Co., Ltd., Tokyo, Japan) at an external laboratory. We converted it to the National Glycohemoglobin Standardization Program (NGSP) equivalent value (%) using the following formula: HbA1c (%) = 1.02×HbA1c (Japan Diabetes Society) (%)+0.25% [24].

### Diagnosis of type 2 diabetes

Type 2 diabetes was diagnosed as a fasting plasma glucose ≥126 mg/dl (7.0 mmol/l), HbA1c ≥6.5% (48 mmol/mol), or under medical treatment for diabetes.

### Statistical analysis

We classified participants into age-specific (18–29, 30–39, 40–49, and ≥50 years) tertiles of estimated VO_2max_ according to previous studies [11]. Means (standard deviation) and percentages of baseline characteristics were compared between subjects with and without incidence of diabetes using t-test for continuous variables and χ^2^ test for categorical variables.

Person-time was calculated from the date of the baseline examination to the date of the first subsequent examination when the diagnosis of diabetes was confirmed or to the date of the last examination, whichever came first. Hazard ratios and 95% confidence intervals of incident diabetes associated with single or combined effects of estimated VO_2max_ and BMI were calculated with the use of Cox proportional-hazards regression models. Trend association was assessed by assigning ordinal numbers 1 to 3 to the highest through the lowest age-specific tertile of estimated VO_2max_. First, we adjusted for age and baseline year. Additionally, we adjusted for smoking status (non-smoker, current smoker consuming 1 to 10, 11–20, or ≥21 cigarettes per day), alcohol consumption (non-drinker or drinker consuming <1 unit, 1 to <2 units, or ≥2 units of alcohol per day, 1 unit of alcohol corresponds to 1 go of Japanese sake which approximately contains 23 g of ethanol), sleep duration (<7, 7 to <9, or ≥9 hours per day), hypertension (presence or absence, defined as a systolic blood pressure ≥140 mmHg, a diastolic blood pressure ≥90 mmHg, or self-reported hospital visit for hypertension treatment), and family history of diabetes (presence or absence) (model 1). We mutually adjusted for BMI (<18.5, 18.5 to <23, 23 to <25, ≥25 kg/m^2^) and fitness (tertile) (model 2). We tested the proportional-hazards assumption using the Schoenfeld residuals and observed no significant deviations for all covariates in model 1.

For joint associations of fitness and BMI, we classified participants into four groups based on their age-specific (the same cutoff as above) fitness level (upper two-thirds or lower one-third) and BMI (<25 or ≥25 kg/m^2^), and treated men with high fit (upper two-thirds of fitness) and normal weight as the reference as a previous study [11] defined. We repeated the analysis after exclusion of participants with follow-up of less than 3 years or those who gained ≥3 kg of weight by within 1 year from baseline. Two-sided P values less than 0.05 were considered statistically significant. All analyses were performed with Stata version 13.1 (Stata Corp, College Station, Texas).

## Results

The mean age of the subjects was 42.2 years (range: 18–61). Overall, 2,881 (81.8%) of men had BMI <25 kg/m^2^, and 642 (18.2%) of men had BMI ≥25 kg/m^2^. Baseline characteristics of men with and without incident T2DM are summarized in Table 1. Compared with participants who did not develop T2DM during follow-up, those who developed were older, had a higher BMI but lower estimated VO_2max_, and tended to smoke, consume alcohol, and have hypertension and family history of diabetes.

**Table 1:** Baseline characteristics of men with and without incident type 2 diabetes mellitus during follow-up.

	Without type 2 diabetes	With type 2 diabetes	P value*
Participants, No.	3,324	199	
Age, mean (SD), year	41.9 (10.5)	47.5 (7.6)	<0.001
BMI, mean (SD), kg/m^2^	22.6 (2.8)	23.8 (3.6)	<0.001
BMI, %			
<18.5 kg/m^2^	5.5	3.5	
18.5 to <25.0 kg/m^2^	77.2	62.8	
25.0 to <30.0 kg/m^2^	15.9	29.7	
≥30.0 kg/m^2^	1.4	4.0	<0.001
Estimated VO_2max_, mean (SD), ml/kg/min	38.5 (7.0)	36.9 (7.2)	0.002
Age-specific tertiles of fitness, %			
Low	33.3	37.7	
Moderate	33.2	37.2	
High	33.5	25.1	0.05
Current smoking, %	34.1	44.7	0.002
Current drinker, %†	21.9	28.1	0.04
Sleeping <7 hours per day, %	54.2	54.3	0.98
Hypertension, %	14.7	29.2	<0.001
Family history of diabetes, %	12.7	18.6	0.016

Abbreviations: BMI, body mass index; SD, standard deviation.

*P value was calculated by t-test for continuous variables and χ^2^ test for categorical variables.

† Consuming ≥2 units of alcohol per day, 1 unit of alcohol corresponds to 1 go of Japanese sake which approximately contains 23 g of ethanol.

During a mean follow-up of 6.0 years and 21,187 person-years of observation, 199 incident cases of T2DM were identified. Table 2 shows the separate association of tertile category of estimated VO_2max_ and BMI (<25 or ≥25 kg/m^2^) with the risk of T2DM. After adjustment for age, entry year, smoking, alcohol consumption, sleep duration, hypertension, and family history of diabetes, the adjusted hazard ratios (95% confidence interval) of developing T2DM were 1.00 (reference), 1.44 (1.01–2.07), and 1.48 (1.03–2.13) for the highest through the lowest tertile of estimated VO_2max_ (P for trend  = 0.04). Additional adjustment for BMI, however, largely attenuated the association of fitness (model 2) (P for trend  = 0.70). For the association of BMI, the adjusted hazard ratio of T2DM in overweight/obese subjects was 2.10 (1.56–2.83) compared to their normal weight counterparts, and additional adjustment for fitness did not appreciably alter the result.

**Table 2:** Hazard ratios with 95% confidence intervals for incident type 2 diabetes mellitus according to age-specific tertile of cardiorespiratory fitness level or weight status

	Fitness			
	High	Middle	Low	P for trend*
Estimated VO_2max_ (ml/min/kg)	44.2 (39.4–93.6)†	37.6 (34.1–43.3)	32.4 (21.1–37.6)	
No. of cases	50	74	75	
Person-years	7,085	7,064	7,039	
Age and baseline year-adjusted	1.00 (reference)	1.48 (1.03, 2.12)	1.51 (1.05, 2.15)	0.029
Multivariable-adjusted model 1‡	1.00 (reference)	1.44 (1.01, 2.07)	1.48 (1.03, 2.13)	0.04
Multivariable-adjusted model 2§	1.00 (reference)	1.26 (0.87, 1.83)	1.10 (0.75, 1.63)	0.70

	BMI				
	<25 kg/m^2^		≥25 kg/m^2^		P value
No. of cases	132		67		
Person-years	17,430		3,757		
Age and baseline year-adjusted	1.00 (reference)		2.27 (1.69, 3.05)		<0.001
Multivariable-adjusted model 1‡	1.00 (reference)		2.10 (1.56, 2.83)		<0.001
Multivariable-adjusted model 2§	1.00 (reference)		2.04 (1.49, 2.80)		<0.001

Abbreviation: BMI, body mass index.

*P for trend was calculated by assigning ordinal numbers 1 to 3 to tertile category of estimated VO_2max_ and treating this variable as continuous.

† Median (range).

‡ Adjusted for age, baseline year (2003, 2004, or 2005), smoking status (non-smoker or current smoker consuming 1 to 10, 11 to 20, or ≥21 cigarettes per day), alcohol consumption (non-drinker or drinker consuming <1 unit, 1 to <2 units, or ≥2 units of alcohol per day, 1 unit of alcohol corresponds to 1 go of Japanese sake which approximately contains 23 g of ethanol), sleep duration (<7, 7 to <9, or ≥9 hours per day), hypertension (presence or absence), and family history of diabetes (presence or absence).

§ Adjusted for factors in model 1 plus BMI (<18.5, 18.5 to <23, 23 to <25, ≥25 kg/m^2^) for fitness, or age-specific tertile category of fitness for BMI.

Table 3 shows the joint associations of estimated VO_2max_ and BMI with risk of developing T2DM. The multivariable-adjusted hazard ratios (95% confidence interval) were 1.00 (reference), 1.32 (0.91, 1.90), 2.94 (1.98, 4.37), and 1.83 (1.21, 2.76) in normal weight high-fit men, normal weight low-fit men, overweight high-fit men, and overweight low-fit men, respectively. The association of fitness with T2DM risk was modified by BMI (P for interaction <0.001). When the analysis was restricted to 3,015 men who gained weight of <3 kg within 1 year of baseline, results were not materially changed (data not shown). After an exclusion of 607 participants with follow-up of less than 3 years (including 111 incident cases of T2DM), the results were slightly changed; the adjusted hazard ratios were 1.00, 1.37, 2.46, and 1.52 in normal weight high-fit men, normal weight low-fit men, overweight high-fit men, and overweight low-fit men, respectively.

**Table 3:** Joint associations of weight status and cardiorespiratory fitness with incident type 2 diabetes mellitus

	BMI <25 kg/m^2^			BMI ≥25 kg/m^2^		
	Fit	Unfit		Fit	Unfit	
	(upper two-thirds)	(lower one-third)	P value*	(upper two-thirds)	(lower one-third)	P value
Estimated VO_2max_						
median (IQR), ml/min/kg	40.7 (37.8–44.5)	32.8 (30.8–34.0)		38.4 (36.4–41.4)	31.5 (29.5–33.3)	
No. of subjects	2,096	785		245	397	
No. of cases	89	43		35	32	
Person-years	12,785	4,646		1,364	2,393	
Age-, sex-, and baseline year-adjusted	1.00 (reference)	1.30 (0.90, 1.87)	0.16	3.26 (2.20, 4.81)	1.93 (1.29, 2.89)	0.033
Multivariable-adjusted model 1†	1.00 (reference)	1.32 (0.91, 1.90)	0.15	2.94 (1.98, 4.37)	1.83 (1.21, 2.76)	0.053

Abbreviations: BMI, body mass index; IQR, interquartile range.

*P value between fit and unfit in each category of BMI.

† Adjusted for age, baseline year (2003, 2004, or 2005), smoking status (non-smoker or current smoker consuming 1 to 10, 11 to 20, or ≥21 cigarettes per day), alcohol consumption (non-drinker or drinker consuming <1 unit, 1 to <2 units, or ≥2 units per day, 1 unit of alcohol corresponds to 1 go of Japanese sake which approximately contains 23 g of ethanol), sleep duration (<7, 7 to <9, or ≥9 hours per day), hypertension (presence or absence), and family history of diabetes (presence or absence).

## Discussion

In the present study among Japanese men, the lowest fit men had higher risk of type 2 diabetes than the highest fit men. This relationship, however, disappeared after adjustment for BMI. Joint analysis showed that overweight/obese high fit men had a higher risk of T2DM than normal weight low-fit men when normal weight high-fit men were treated as the reference. This is the first study to investigate the association of fitness with diabetes risk using HbA1c, and one of the few studies examining the joint associations of fitness and BMI with incident T2DM.

The present finding of no association of fitness with diabetes risk after adjustment for BMI suggests that the effects of fitness against type 2 diabetes are largely mediated by body weight. This finding is inconsistent with those from the ACLS using treadmill maximal exercise test in women [11] and men [12] and the Tokyo Gas Study in Japanese men using a submaximal exercise test on a cycle ergometer [15], showing a significant inverse association even after adjustment for BMI. One reason for this discrepancy may be ascribed to the difference in fitness assessment; the maximal workload in our study was set up to 70% of age- and sex-specific estimated maximum heart rate, whereas it was 85% of the age-specific maximal heart rate in the Tokyo Gas Study [15] and subjects were encouraged to give maximal effort (100%) in the treadmill exercise test in the ACLS [11], [12]. The lower maximal workload in our study might have caused greater misclassification in the fitness level, leading to an attenuation of risk associated with fitness. In fact, in a model that did not adjust for BMI, subjects with high fitness had a 32% lower risk of diabetes than those with low fitness in the present study, whereas a greater risk reduction (51 to 75%) was observed in previous studies [11], [12], [15]. Alternatively, our mean follow-up period (6 years) was shorter than those in the ACLS (17 years in women [11] and 7 years in men [12]) and the Tokyo Gas Study (14 years) [15]. The effect of fitness might be apparent only after a long period of follow-up.

We observed that, compared with normal weight high-fit men, normal weight low-fit men had a 1.3 times higher risk of T2DM, whereas overweight/obese (BMI ≥25 kg/m^2^) high-fit men had a 2.9 times higher risk of T2DM. This result suggests a greater importance of weight control than fitness in type 2 diabetes prevention. Of two reports from the ACLS on this issue [11], [12], our results are in agreement with the report in women [11] that showed a higher risk of T2DM among overweight/obese (BMI ≥25 kg/m^2^) high-fit women than that of normal weight low-fit women. The report in men [12] showed that obese (BMI ≥30 kg/m^2^) high-fit men had a higher risk of T2DM than overweight (BMI 25 to <30 kg/m^2^) low-fit men, but both normal weight low-fit men and overweight high-fit men had a similar risk of T2DM compared with normal weight high-fit men. The reason for the discrepancy among studies may be ascribed to the difference in the cutoff for BMI, method of fitness assessment, and length of follow-up period.

Several plausible mechanisms are suggested for an inverse association of regular physical activity with risk of type 2 diabetes. Physical activity helps to reduce body weight [25], and to maintain/increase skeletal muscle mass, and to decrease adipose tissue mass [26]. Accumulating evidence [27] has shown that adipose tissue releases increased amounts of hormones, proinflammatory cytokines, non-esterified fatty acids, and glycerol, which are all involved in the development of insulin resistance, a key precursor of T2DM. Additionally, recent studies [28], [29] have shown that an increase in physical activity followed by weight loss improves insulin secretory capacity. Independent of the effects of physical activity on body weight and composition, physical activity may directly improve insulin sensitivity [26].

The strengths of our study include a large population size, a prospective design, and assessment of T2DM using annual fasting plasma glucose and HbA1c levels. HbA1c has several advantages over fasting glucose, including that it is a better index of overall glycemic exposure, is relatively less prone to acute perturbations in glucose levels, and has substantially less biological variability and pre-analytic instability [20]. Thus, the combined use of fasting glucose and HbA1c criteria would more precisely detect cases with T2DM than the use of a fasting glucose criterion alone [30]. The limitations of our study should be mentioned. First, cardiorespiratory fitness was not evaluated using directly measured VO_2max_ with a maximal exercise test on a treadmill or bicycle ergometer, an ideal measurement of this fitness [31]. Second, our subjects were employees of a large company, and it is thus difficult to generalize our findings to employees of small to medium-sized companies and the general population. Finally, information on the diet was not available in this study. However, we adjusted for putative and potential confounders that may influence T2DM risk.

We found that overweight/obese and high fit men had higher risk of T2DM than normal weight and low-fit men in Japanese men, suggesting a greater importance of keeping normal weight than improving physical fitness in the prevention of type 2 diabetes. Nevertheless, because engaging in physical activity is an effective strategy for weight management, it remains an important component of type 2 diabetes prevention.
